# Preventing and Repairing Myeloma Bone Disease by Combining Conventional Antiresorptive Treatment With a Bone Anabolic Agent in Murine Models

**DOI:** 10.1002/jbmr.3606

**Published:** 2018-11-26

**Authors:** Julia Paton‐Hough, Simon Tazzyman, Holly Evans, Darren Lath, Jenny M Down, Alanna C Green, John A Snowden, Andrew D Chantry, Michelle A Lawson

**Affiliations:** ^1^ Sheffield Myeloma Research Team Department of Oncology and Metabolism, Medical School University of Sheffield Sheffield UK; ^2^ Mellanby Centre for Bone Research University of Sheffield Medical School, University of Sheffield Sheffield UK; ^3^ Department of Haematology Sheffield Teaching Hospitals NHS Foundation Trust Royal Hallamshire Hospital Sheffield UK

**Keywords:** MULTIPLE MYELOMA, ZOLEDRONIC ACID, BONE ANABOLIC THERAPY, BONE LESION REPAIR, ANTI‐TGFβ/1D11

## Abstract

Multiple myeloma is a plasma cell malignancy, which develops in the bone marrow and frequently leads to severe bone destruction. Current antiresorptive therapies to treat the bone disease do little to repair damaged bone; therefore, new treatment strategies incorporating bone anabolic therapies are urgently required. We hypothesized that combination therapy using the standard of care antiresorptive zoledronic acid (Zol) with a bone anabolic (anti‐TGFβ/1D11) would be more effective at treating myeloma‐induced bone disease than Zol therapy alone. JJN3 myeloma‐bearing mice (*n* = 8/group) treated with combined Zol and 1D11 resulted in a 48% increase (*p* ≤ 0.001) in trabecular bone volume (BV/TV) compared with Zol alone and a 65% increase (*p* ≤ 0.0001) compared with 1D11 alone. Our most significant finding was the substantial repair of U266‐induced osteolytic bone lesions with combination therapy (*n* = 8/group), which resulted in a significant reduction in lesion area compared with vehicle (*p* ≤ 0.01) or Zol alone (*p* ≤ 0.01). These results demonstrate that combined antiresorptive and bone anabolic therapy is significantly more effective at preventing myeloma‐induced bone disease than Zol alone. Furthermore, we demonstrate that combined therapy is able to repair established myelomatous bone lesions. This is a highly translational strategy that could significantly improve bone outcomes and quality of life for patients with myeloma. © 2018 The Authors. *Journal of Bone and Mineral Research* Published by Wiley Periodicals Inc.

## Introduction

Multiple myeloma is a cancer of plasma cells that frequently causes severe bone destruction and debilitating bone pain, resulting in substantially diminished functional capacity and quality of life.^1^, ^2^ Across a retrospective study, approximately 80% of all myeloma patients experienced pathological fractures and 90% had osteolytic bone lesions over the course of disease,^3^ which correlates with poor survival and mortality.^4^ Myeloma‐induced bone disease occurs via several mechanisms. In most cases, osteoclast‐stimulating factors including receptor activator of NF‐κB ligand (RANKL),^5^, ^6^ macrophage inflammatory protein 1,^7^, ^8^ and various interleukins (IL‐1β,^9^ IL‐3,^10^ and IL‐6^11^) are produced by myeloma cells or tumor‐activated cells in the bone marrow microenvironment (BMME), causing an increase in bone resorption. In addition, osteoblast inhibitors such as dickkopf‐1 (Dkk‐1),^12^, ^13^ activin A,^14^, ^15^ and sclerostin^16^, ^17^ are upregulated in patient sera resulting in a decrease in bone formation. Overall, this imbalance of bone remodeling results in severe bone loss. Myeloma patients presenting with bone disease are commonly prescribed the antiresorptive agent zoledronic acid (Zol).^18^ However, although Zol and other antiresorptives are effective at inhibiting osteoclastic bone resorption, they do virtually nothing to repair existing bone damage and as a result, bone remains weak and at risk of fracture.

Bone anabolic therapies using inhibitors of activin A^15^ and Dkk‐1^19^, ^20^ have shown promise in preclinical models of myeloma but thus far have resulted in only modest benefit in clinical trials^21^, ^22^, ^23^ and have also led to some unexpected side effects. For example, rises in hematocrit after treatment with Sotatercept, a decoy receptor for activin A, have been observed, leading to a repurposing of this and similar agents as a treatment for cancer‐induced anaemia.^21^ More recently, deletion of the sclerostin gene (*Sost*) or administration of an anti‐sclerostin antibody prevented bone disease in murine models of myeloma.^24^ Furthermore, when an anti‐sclerostin treatment was used in combination with Zol in preclinical models of myeloma, using a preventative regimen, they showed superior effects on fracture resistance compared with Zol alone.^25^ However, sclerostin's effectiveness on established osteolytic bone disease has not yet been assessed in murine models or in patients with myeloma. In osteoporotic patients, anti‐sclerostin therapy (Romosozumab) was associated with a lower risk of fracture^26^ but was also associated with potential adverse cardiac events.^27^ In addition, the positive effects of anti‐sclerostin therapy upon bone formation were only sustained for a short period.^26^ Furthermore, a combination of Dkk‐1 and sclerostin inhibition using a bi‐specific antibody approach demonstrated great promise at increasing bone formation and bone strength in naïve rodents and therefore would be a desirable method to test in preclinical models of myeloma.^28^

As indicated, there is a clear demand for new bone anabolic agents for patients with myeloma and established osteolytic bone disease. It is hoped that such therapies will enhance bone formation for sustained periods, leading to repair of damaged bones. This has clear translational potential for substantially improving the morbidity and quality of life in terms of fracture risk, pain control, and functional status for patients with myeloma bone disease.

A potential therapeutic target in myeloma is TGFβ, which is a cytokine reported to have both inhibitory^29^, ^30^ and stimulatory^31^, ^32^ roles in bone formation. Specifically, it is known to inhibit osteoblast differentiation. TGFβ is also known to be expressed by patient myeloma cells,^33^, ^34^ is upregulated in sera from some monoclonal gammopathy of undetermined significance (MGUS) patients,^35^ and is associated with immunoparesis in myeloma patients at various stages of the disease.^36^ It has also been shown to be overexpressed by bone marrow stromal cells (BMSC) from myeloma patients^34^ and is liberated from bone mineral matrix undergoing resorption. TGFβ's actions can be blocked using a TGFβRI inhibitor (SD‐208) in vitro^37^ and, more recently, TGFβ inhibition has been used as anti‐tumor therapy in preclinical models of myeloma,^38^, ^39^ breast cancer,^40^, ^41^, ^42^ and prostate cancer.^43^ Interestingly, several of these inhibitors are now in cancer clinical trials as anti‐tumor drugs (e.g. NCT02452008 and NCT01401062^44^, ^45^). However, TGFβ inhibition has been shown to have direct bone anabolic effects in naïve animals, resulting in increased bone formation and bone integrity.^29^ This complements findings from immune‐competent models of myeloma where TGFβ inhibition resulted in significantly higher vertebral strength and fewer femoral osteolytic lesions compared with control mice.^46^ Currently, no studies have explored the efficacy of bone anabolic therapy in combination with an antiresorptive specifically to evaluate whether repair of existing osteolytic lesions is possible (previous studies have only focused on preventing the development of bone lesions at the end stage of disease). Here we show that bone anabolic therapy using an anti‐TGFβ (1D11) antibody combined with the standard of care antiresorptive Zol are able to repair existing osteolytic lesions and are more effective at repairing osteolytic lesions and preventing myeloma bone disease than either therapy alone.

## Materials and Methods

### Ethics statement

All procedures involving animals were conducted at the University of Sheffield (UK) and were approved by the Home Office (PPL 70/8799) and the University of Sheffield's Animal Ethics Committee in accordance with the Animal (Scientific Procedures) Act 1986.

### Osteoblast isolation and differentiation

Primary calvarial osteoblast‐like cells (Ob‐LC) were isolated and differentiated as described previously^47^ using 4% FCS and BMP‐2 (30 ng/mL) (Bio‐Techné, Abingdon, UK). To determine the effect of TGFβ upon osteoblast differentiation, Ob‐LC were cultured with either vehicle (CTRL), recombinant TGFβ1 (rTGFβ1) (5 ng/mL) and mouse IgG1 isotype control (IC) antibody (25 μg/mL) (Bio‐Techné) (rTGFβ + IC), or rTGFβ1 (5 ng/mL) and 1D11 antibody (25 μg/mL) (Bio‐Techné), (rTGFβ + 1D11). Media was changed every 2 to 3 days and differentiation was assessed at 7 and 14 days after treatment began by examining alkaline phosphatase (Alp) and at 14 and 21 days using alizarin red staining to detect mineralization, as described previously.^48^ Osteosarcoma SAOS‐2 cells (ATCC, Teddington, UK) were cultured as described previously,^49^ treated as above for 3 days, and cultured in 1% FCS to measure Alp. Each experiment was repeated at least three times (*n* = 3) and representative data are presented as a fold change.

### Osteoclast assays

Bone marrow osteoclasts were isolated and cultured from 4‐week‐old female BALB/c mice as previously described.^50^ Cells were then treated with either vehicle (CTRL), IC (25 μg/mL), or 1D11 (25 μg/mL) every 2 to 3 days for 14 days. Osteoclast numbers and resorption area were then analyzed as previously described.^50^

### Myeloma cell viability assays

JJN3 and U266 human myeloma cell lines were cultured in RPMI‐1640 medium (containing 10% FCS, 1% penicillin/streptomycin, 100 U/100 µg/mL, 1% non‐essential amino acids, and 1% sodium pyruvate, 1 mM) at 37°C in 5% CO_2_. Cells were seeded (at 2 × 10^4^ cells/well) in triplicate in 96‐well plates and treated with either vehicle (CTRL), IC (25 μg/mL), or 1D11 (25 μg/mL). After 4 hours, 24 hours, 48 hours, and 72 hours, cell viability was measured using an Alamar Blue assay (ThermoFisher, Warrington, UK) according to the manufacturer's instructions.

### ELISA

ELISA was used to detect murine TRAP5b (Oxford Biosystems, Oxford, UK), murine P1NP (Immuno Diagnostic Systems, Tyne & Wear, UK), murine IL‐6 (Abcam, Cambridge, UK), and human IgE paraprotein (ThermoFisher) in murine sera following the manufacturer's instructions.

### In vivo studies

Nod/Scid γ (NSG, NOD.Cg‐Prkdcscid Il2rgtm1Wjl/SzJ) mice were purchased from Charles River Laboratories (UK). Animal numbers were calculated using G*Power software recommended by NC3Rs. All animals were housed in the Biological Service Unit at the University of Sheffield in individual ventilated cages. Both an aggressive myeloma model using JJN3 cells and a moderately aggressive model using U266 cells were used in the in vivo experiments. In NSG mice, these cell types specifically colonize the bone after intravenous (iv) injection with no extramedullary growth resulting in bone disease as described previously.^51^, ^52^, ^53^

### The effect of 1D11 in naïve and JJN3‐bearing NSG mice

Seven‐ to 8‐week‐old female NSG mice were randomized into groups (*n* = 5/group) and injected iv via the tail vein with 100 µL PBS (Naïve) or 1 × 10^6^ JJN3 cells (JJN3). After 7 days, mice were treated with either 20 mg/kg IgG1 IC (Naïve + IC/ JJN3 + IC) or 20 mg/kg 1D11 (Naïve + 1D11/ JJN3 + 1D11) via intraperitoneal (ip) injection every 3 days and mice were culled after 21 days post‐tumor cell injection.

Mouse group numbers were ascertained through the use of the power calculation formula:

$${2(SD)}^{2}\times f\left(\alpha ,\beta \right)/\Delta^{2}$$

where α (significance level) was 0.05, β (power level) was 90%, and both Δ (least practicable difference between groups) and standard deviation were taken from a similar study with regard to percentage trabecular bone volume (BV/TV, bone volume/total volume). It gave rise to the following power calculation:

$${2\left(1.4\right)}^{2}\times 10.5/3^{2}=4.6,\;\;i.e.\;\text{ 5 mice.}$$

### Assessment of tumor burden and bone disease (ex vivo)

At the end of the experiment, mice were euthanized and the bone marrow from the left femora were flushed and tumor burden was assessed by anti‐human leukocyte antigen (HLA) staining analyzed by flow cytometry using a FACS Calibur and Cell Quest software (BD Biosciences San Jose, CA, USA) as described previously.^54^ Tumor burden was also quantified in hematoxylin‐stained paraffin‐embedded sections, which were scanned using a Hamamatsu Nanozoomer XR (Hamamatsu City, Japan) and analyzed by morphology as a percentage of the bone marrow using Image Scope software (Leica Biosystems, Buffalo Grove, IL, USA). To assess bone disease, after mouse euthanasia, the right and left tibias were analyzed by μCT using a SkyScan 1272 ex vivo μCT scanner at 50 kilovolts (kV) and 200 microamperes (μA), using an aluminium filter of 0.5 mm and pixel size of 4.3 μm^2^ as described previously.^19^, ^51^ BV/TV, trabecular number, trabecular thickness, cortical thickness, lesion area and lesion number parameters were then assessed as described previously^51^ and according to standard guidelines.^55^ 3D models of trabecular bone were created using ParaView Software (Clifton Park, NY, USA). Histomorphometry was used to detect differences in osteoblast and osteoclast numbers on longitudinal bone sections using Osteomeasure software (Osteometrics, Decatur, GA, USA), as described previously^51^ and following standard guidelines.^56^

### Efficacy of 1D11 and Zol combination therapy in the JJN3 murine model of MM

Eight‐ to 9‐week‐old female mice were randomized into groups (*n* = 8/group) and treated as follows: group 1, PBS iv (Naïve); group 2, 1 × 10^6^ JJN3 cells iv and IC (20 mg/kg IgG1, ip every 3 days post‐tumor cell injection) (JJN3); group 3, JJN3 cells and Zol (Procter & Gamble, 125 µg/kg subcutaneously (sc) on days 13 and 16 post‐tumor cell injection) and IC (Zol); group 4, JJN3 cells and 1D11 (20 mg/kg, ip every 3 days post‐tumor cell injection) (1D11); or group 5, JJN3 cells, Zol, and 1D11 (Combo). After 21 days post‐tumor cell injection, all animals were euthanized. Tumor burden and bone disease were assessed as described above. Murine P1NP, TRAP5b, and IL‐6 serum levels were quantified by ELISA as described above. Mouse group numbers were determined using the same power calculation formula shown above. α was taken as 0.05, β was taken as 90%, and Δ and SD values were taken from the results of the “effect of 1D11 in naïve and JJN3‐bearing NSG mice” study described above with regard to percentage BV/TV. It gave rise to the following power calculation:

$${2\left(1.8\right)}^{2}\times 10.5/3^{2}=7.6,\;\;ie,\;\;8\;\;mice$$

### Longitudinal monitoring of lesion repair after 1D11 and Zol combination therapy in the U266 murine model of myeloma

Twenty‐two female NSG mice (7 to 8 weeks old) were injected iv with 1 × 10^6^ U266 cells. At 5 weeks post‐tumor cell injection, bone disease progression was monitored twice a week in the right tibias of each mouse using a VivaCT 80 in vivo preclinical μCT scanner (Scanco, Zurich, Switzerland) as described below. Upon detection of established osteolytic lesions (at approximately 6 weeks post‐U266 injection), mice were randomly split into three groups and treated as follows: group 1, vehicle (U266); group 2, Zol (125 µg/kg sc, 3 days apart) and IC (20 mg/kg IgG1, ip, every 3 days) (Zol); or group 3, 1D11 (20 mg/kg, ip every 3 days) and Zol (Combo). Mice were culled 3 weeks post‐treatment (group 1, *n* = 6; group 2, *n* = 8; and Group 3, *n* = 8) and tumor burden was assessed by flow cytometry (HLA staining), histology (morphology), and ELISA (IgE paraprotein levels) as described above.

Mouse group numbers were determined using the same power calculation shown above:

$${2\left(1.8\right)}^{2}\times 10.5/3^{2}=7.6,\;\;ie,\;\;8\;\;mice.$$

However, because of a smaller cohort than anticipated, only 6 mice were available for the vehicle group. This was deemed acceptable because the vehicle group was less variable than the treatment groups with regards to bone lesion development, based on previously published data.^51^

### Bone disease assessment (in vivo)

Mice were anesthetized and right tibias were scanned using a VivaCT 80 at 45 kV and 177 µA with a voxel size of 10.4 µm on week 0 (W0; when bone disease was first detected after tumor cell inoculation, at approximately 6 weeks) and then again after 3 weeks (W3). For image registration, W3 tibia data sets were registered to their paired W0 control data sets using MIM Maestro (v. 6.6.6, Cleveland, OH, USA). After registration, both data sets were volumetrically registered using Drishti software (v. 10, ANU Vizlab, Acton, Australia) and quantified using ImageJ (version 1.47, NIH, Bethesda, MD, USA). The percentage change in lesion area, cortical thickness, and BV/TV was then calculated from time of disease onset (W0) to 3 weeks post‐treatment (W3), presented as the mean percentage change for each group. 3D transverse models of the tibias were created using ParaView software and 3‐D longitudinal models of the tibias were created using Drishti software.

### Statistical analysis

All data were analyzed using GraphPad Instat version 6.0b (La Jolla, CA, USA). Where possible, the distribution of data was analyzed using a D'Agostino‐Pearson omnibus normalization test and relevant parametric or nonparametric statistical tests were used. If normalization was not possible, normal distribution was assumed and data were analyzed using either a Student's *t* test or one‐way ANOVA with a Tukey's multiple comparison test. Where data was not normally distributed, a nonparametric Kruskal‐Wallis test followed by a Dunn's multiple comparison test was used. Any potential outliers were identified using Grubb's outlier analysis and removed accordingly. All data were expressed with error bars representing mean ± standard deviation (SD).

### Data sharing

Data will be accessible to the public after publication via ORDA, the University of Sheffield's Research Data Catalogue and Repository. All data are preserved for 10 years and those interested in obtaining access to these data must contact the first author via email.

## Results

### TGFβ inhibition restores osteoblast differentiation in vitro and has a bone anabolic effect in vivo

TGFβ, as described above, is secreted by patient myeloma cells and BMSC^34^ and is also liberated from bone matrix upon resorption.^57^ Importantly, TGFβ is also an inhibitor of osteoblast differentiation, thus contributing to the mechanism of bone disease in myeloma patients. Therefore, we wanted to, first, confirm that blocking TGFβ using a monoclonal anti‐TGFβ antibody (1D11) would prevent rTGFβ1 inhibition of osteoblast differentiation and, second, that 1D11 would result in a bone anabolic effect in naïve and tumor‐bearing mice.

**Figure 1:**
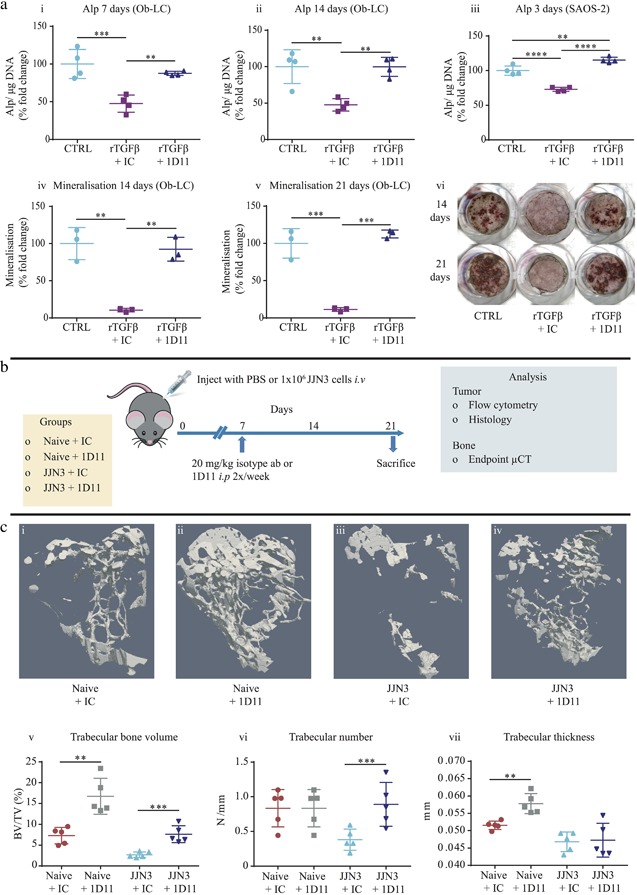
**Administration of anti‐TGFβ (1D11) prevented rTGFβ inhibition of osteoblastic alkaline phosphatase production and mineralization in vitro and resulted in a substantial bone anabolic effect in vivo.** (*A*) Alp production in primary murine Ob‐LC cultured in osteogenic media with vehicle (CTRL), rTGFβ and IC antibody (rTGFβ + IC), or rTGFβ and 1D11 antibody (rTGFβ + 1D11) for 7 (i) and 14 days (ii). Alp production in SAOS‐2 cells cultured in standard media and treated as above for 3 days (iii). Mineralization in Ob‐LC treated as above for 14 (iv) and 21 days (v). Representative images of mineralization in Ob‐LC treated as above (vi). All data are representative from four independent experiments, presented as mean fold change ± SD, one‐way ANOVA, **p ≤ 0.01, ***p ≤ 0.001, and ****p ≤ 0.0001. (*B*) Schematic demonstrating the treatment schedule for 1D11 monotherapy treatment in Naïve mice and the JJN3 model of myeloma. (*C*) Representative 3D trabecular μCT images from the tibias of Naïve + IC (i), Naïve + 1D11 (ii), JJN3 + IC (iii), and JJN3 + 1D11 (iv) mice. BV/TV (v), trabecular number (vi), and trabecular thickness (vii) analyzed in the tibias by μCT. All data are presented as mean ± SD, Student's *t* test, **p ≤ 0.001 and ***p ≤ 0.001.

Treatment of primary murine calvarial Ob‐LC with rTGFβ1 resulted in a 52% (*p* ≤ 0.001) and 58% (*p* ≤ 0.01) reduction in Alp at 7 and 14 days, respectively, which was prevented upon treatment with 1D11 at both time points (*p* ≤ 0.01) (Fig. 1 *A* i–ii). This effect was also observed using the human osteosarcoma cell line SAOS‐2 (Fig. 1 *A* iii). In addition, rTGFβ1 inhibited Ob‐LC mineralization by 89% at 14 days (*p* ≤ 0.01) and 88% at 21 days (*p* ≤ 0.001); this was also prevented after treatment with 1D11 (*p* ≤ 0.01, *p* ≤ 0.001, respectively) (Fig. 1 *A* iv–vi). This confirmed similar results reporting that TGFβ prevents osteoblast differentiation in MC3T3‐E1, C2C12 cells, and primary BMSC from myeloma patients, an effect that could be blocked by TGFβ inhibition.^39^, ^58^

We then verified the translational efficacy of this concept in vivo by administering 1D11 into naïve mice and mice bearing JJN3 myeloma cells (treatment schedule described in Fig. 1 *B*). 1D11 treatment in vivo resulted in a significant increase in BV/TV in both naïve (130%) (*p* ≤ 0.01) and JJN3‐bearing mice (183%) (*p* ≤ 0.001) compared with IC‐treated animals (Fig. 1 *C* i–v). JJN3‐bearing mice treated with 1D11 also had a higher trabecular number (Fig. 1 *C* vi) (*p* ≤ 0.01), whereas naïve, but not JJN3‐bearing, mice treated with 1D11 had thicker trabeculae (Fig. 1 *C* vii) (*p* ≤ 0.01). These data confirmed previous reports that 1D11 was a bone anabolic agent in naïve mice^29^ and prevented trabecular bone loss in immune‐competent myeloma‐bearing animals.^46^

### TGFβ inhibition (1D11) in combination with Zol significantly increased trabecular bone volume compared with monotherapy with either agent in the JJN3 model of myeloma

**Figure 2:**
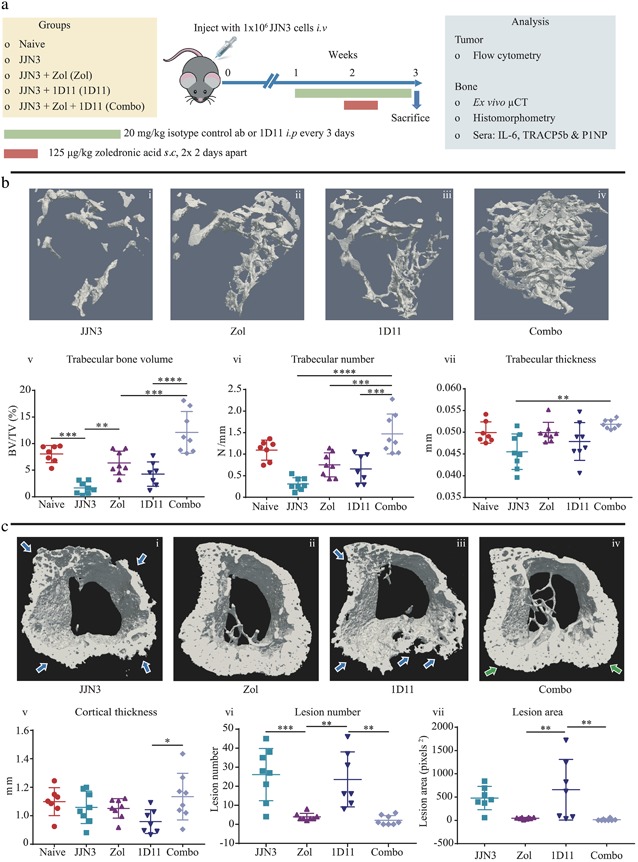
**Administration of anti‐TGFβ (1D11) in combination with Zol significantly increased BV/TV compared with monotherapy in the JJN3 model of myeloma.** (*A*) Schematic demonstrating the treatment schedule for Zol or 1D11 monotherapies or in combination in the JJN3 model of myeloma. (*B*) Representative 3D trabecular bone μCT images from the tibias of JJN3 + vehicle (JJN3) (i), JJN3 + Zol (Zol) (ii), JJN3 + 1D11 (1D11) (iii), and JJN3 + 1D11 + Zol (Combo) (iv) mice. BV/TV (v), trabecular number (vi), and trabecular thickness (vii) analyzed in the tibias by μCT. (*C*) Representative 3D transverse cortical and trabecular bone μCT images from the tibias of JJN3 (i), Zol (ii), 1D11 (iii), and Combo (iv) mice; blue arrows show areas of bone loss and green arrows show areas of new bone formation. Cortical thickness (v), lesion number (vi), and lesion area (vii) analyzed in the tibias by μCT. All data are presented as mean ± SD, one‐way ANOVA and Tukey's multiple comparison test, *p ≤ 0.05, **p ≤ 0.01, ***p ≤ 0.001, and ****p ≤ 0.0001.

Because 1D11 promoted bone formation in naïve mice (Fig. 1 *C* i–ii) and prevented bone loss in JJN3‐bearing mice (Fig. 1 *C* iii–iv), we investigated whether administration with the antiresorptive Zol would further enhance the bone anabolic effect of 1D11 in the JJN3 model of myeloma (treatment schedule described in Fig. 2 *A*). Zol alone significantly increased BV/TV (*p* ≤ 0.01) compared with tumor control (Fig. 2 *B* i–ii, v) (confirming previous findings^51^). Similarly, 1D11 prevented bone loss compared with the tumor control group (Fig. 2 *B* i, iii, v), confirming our previous data (Fig. 1 *C* iii–v). Most interestingly, when Zol and 1D11 were given in combination, BV/TV was 48% higher (*p* ≤ 0.001) compared with Zol (Fig. 2 *B* ii, iv, v) and 65% higher (*p* ≤ 0.0001) compared with 1D11 alone (Fig. 2 *B* iii, iv, v). The trabecular number was also significantly higher in the combination group compared with Zol alone (Fig. 2 *B* vi) (*p* ≤ 0.001), whereas there were no significant changes in thickness of the trabeculae between the Zol, 1D11, or the combination groups (Fig. 2 *B* vii). In contrast to trabecular bone, cortical bone thickness was comparable between naïve and JJN3‐bearing mice (Fig. 2 *C* v), indicating JJN3 tumor cells did not cause significant loss of cortical bone. Zol and 1D11 monotherapies had no effect on cortical thickness compared with vehicle controls. However, cortical thickness was higher in mice receiving combination therapy compared with 1D11 alone (Fig. 2 *C* iii, iv, v). Analysis of osteolytic lesions in the cortical bone revealed vehicle‐treated JJN3‐bearing mice had an average of 26 ± 13.8 lesions in the area analyzed (Fig. 2 *C* vi), and 1D11 monotherapy had no effect on lesion number or size compared with the JJN3 with vehicle (Fig. 2 *C* vi, vii). In comparison, administration of Zol over 2 weeks was highly effective at preventing osteolytic lesion development in mice, with only 4 ± 1.9 lesions present (Fig. 2 *C* vi). Similarly, mice treated with Zol and 1D11 in combination had virtually no cortical bone lesions, with only 2 ± 2.5 lesions per mouse in the area analyzed (Fig. 2 *C* vi). Although osteolytic lesion number and size were slightly lower in mice treated with the combination compared with Zol treatment alone, this was not significant because Zol treatment alone prevented the development of virtually all lesions in mice.

Overall, this study clearly shows that TGFβ inhibition therapy when combined with Zol was more effective at preventing trabecular bone loss than either monotherapy alone in an aggressive murine model of myeloma.

**Figure 3:**
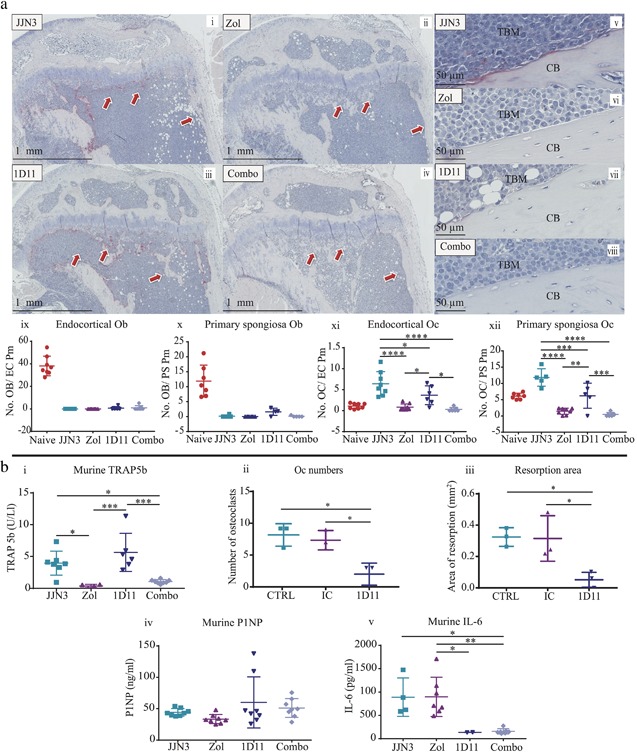
**Zol and 1D11 combination therapy did not significantly increase bone formation or decrease bone resorption compared with monotherapies in the JJN3 model of myeloma.** (*A*) Representative histological images of tibia sections from JJN3‐bearing mice treated with IC (i, v), Zol (ii, vi), 1D11 (iii, vii), and Combo (iv, viii), at ×20 magnification (i–iv) and ×40 magnification (v–viii) showing tumor bone marrow (TBM) and cortical bone (CB). Number of osteoblasts on the endocortical surface (ix) and on the bone primary spongiosa (x). Number of TRAP‐positive osteoclasts on the endocortical surface (xi) and on the primary spongiosa (xii). (*B*) Murine TRAP5b (i) present in murine sera when detected by sandwich ELISA. Primary murine osteoclast numbers (ii) and resorption area (iii) after treatment with vehicle (CTRL), IC, or 1D11 after 3 days. Murine P1NP (iv) and murine IL‐6 (v) present in murine sera when detected by sandwich ELISA. All data are presented as mean ± SD, one‐way ANOVA, *p ≤ 0.05, **p ≤ 0.01, ***p ≤ 0.001 and ****p ≤ 0.0001.

To determine whether these observations were mediated via a bone anabolic or antiresorptive effect, histological analysis was conducted by quantifying osteoblast and osteoclast numbers on endocortical and trabecular bone surfaces in the primary spongiosa. Unexpectedly, when osteoblast numbers were analyzed, there were no significant differences between any of the tumor groups looking at the end stage of disease (Fig. 3 *A* i–x). However, 1D11 treatment did result in fewer osteoclasts on endocortical bone surfaces (42%) (*p* ≤ 0.05) and in the primary spongiosa region (47%) (*p* ≤ 0.0001) (Fig. 3 *A* i, iii, v, vii, xi, xii), which was more pronounced with Zol treatment and resulted in even fewer osteoclasts on the endocortical surface (85%) (*p* ≤ 0.0001) and primary spongiosa (88%) (*p* ≤ 0.0001) compared with JJN3 IC‐treated animals (Fig. 3 *A* i, ii, v, vi, xi, xii). In mice receiving combination treatment, trabecular and endocortical osteoclast numbers were comparable to monotherapy of Zol with no further reduction in osteoclast numbers (Fig. 3 *A* ii, iv, vi, viii, xi, xii), although because Zol alone reduced osteoclast numbers to effectively zero, it is impossible to ascertain whether there would have been a further reduction with the combination treatment. To further investigate the effect of combination therapy on bone resorption and formation, systemic analysis of bone turnover markers TRAP5b and P1NP in mouse sera were also conducted at the end stage of disease. TRAP5b concentrations in Zol‐treated animals correlated with the histological osteoclast data, whereby Zol alone significantly reduced TRAP5b levels compared with tumor (*p* ≤ 0.05), but this was not observed in the 1D11 alone group (Fig. 3 *B* i). Therefore, we assessed the effects of 1D11 on primary murine osteoclasts in vitro and observed a significant reduction in their numbers (Fig. 3 *B* ii) and resorption area (Fig. 3 *B* iii) compared with controls (vehicle and IC). However, despite this, TRAP5b serum levels in the in vivo study were not further reduced in the combination treatment group compared with Zol alone, which was consistent with the histological osteoclast data. P1NP levels also correlated with the histological osteoblast data, where at the end stage of disease, circulating levels were unchanged between all groups (Fig. 3 *B* iv).

We sought to understand why osteoclast numbers were decreased by ID11 treatment and chose to investigate the role of IL‐6. Previously, IL‐6 has been shown to be upregulated in myeloma patient sera^59^ and by BMSC, particularly when in contact with myeloma cells,^57^ and IL‐6 is thought to increase the proliferation/differentiation of osteoclasts.^60^ TGFβ is also known to upregulate the production of IL‐6 by BMSC, the effect of which can be blocked using a TGFβ signaling antagonist (SD‐208, a small molecule inhibitor).^37^ Therefore, TGFβ in myeloma is also likely to contribute to bone disease by upregulation of IL‐6 in the BMME. Consequently, we investigated IL‐6 in the sera of mice at the end stage of disease and found that IL‐6 levels were unchanged in tumor control or Zol‐treated mice, but were significantly reduced in mice treated with either 1D11 alone or 1D11 in combination with Zol (*p* ≤ 0.05 and *p* ≤ 0.001, respectively) (Fig. 3 *B* v). It is therefore suggested that this IL‐6 reduction was specific to the 1D11 treatment with no additive or synergistic effect with Zol.

Overall, using the JJN3 model of MM, combination therapy showed a clear additive effect upon increasing trabecular bone. However, limitations of this 3‐week model of myeloma, ie, the rapidly progressive osteolytic phenotype, made it difficult to identify if combination therapy was more effective than monotherapy at protecting against or repairing myeloma bone disease in the cortical bone. We therefore chose to investigate the effects of combination therapy in the U266 model of myeloma, which also features a marked destructive bone phenotype but develops less quickly over a more protracted period (after 6 to 9 weeks post‐tumor cell injection).

### Established U266‐induced osteolytic lesions are repaired with combined TGFβ inhibition (1D11) and Zol therapy

**Figure 4:**
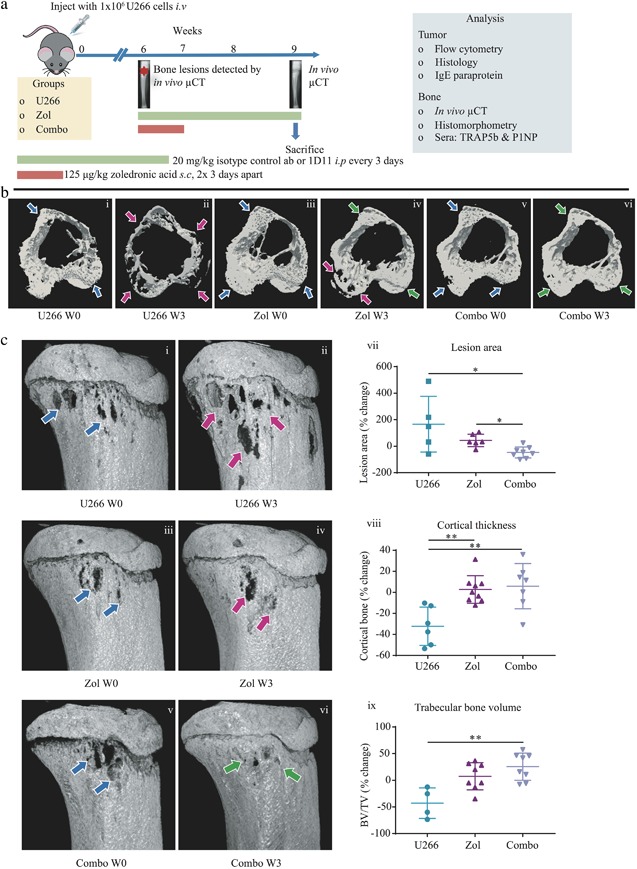
**Zol and 1D11 combination therapy repaired osteolytic bone lesions in the U266 murine model of myeloma.** (*A*) Schematic demonstrating the treatment schedule for Zol monotherapy or Zol and 1D11 combined (Combo) in the U266 model of myeloma. (*B*) W0: Time of bone disease presentation (approximately 6 weeks post‐U266 injection). W3: 3 weeks after treatment. Representative 3D transverse cortical and trabecular registered in vivo μCT images from the tibias of U266 vehicle W0 (U266 W0) (i), U266 vehicle W3 (U266 W3) (ii), U266 + Zol W0 (Zol W0) (iii), U266 + Zol W3 (Zol W3) (iv), U266 + Combo W0 (Combo W0) (v), and U266 + Combo W3 (Combo W3) (vi) mice. (*C*) Representative longitudinal in vivo μCT images from the tibias of U266 W0 (i), U266 W3 (ii), Zol W0 (iii), Zol W3 (iv), Combo W0 (v), and Combo W3 (vi) mice. Blue arrows represent initial bone disease, pink arrows represent progressive bone disease, and green arrows represent a reduction in bone disease and lesion repair. Percentage change in lesion area (vii) from W0 to W3 by in vivo μCT, after image registration. Percentage change in cortical thickness (viii) from W0 to W3 by in vivo μCT. Percentage change in BV/TV (ix) from W0 to W3 by in vivo μCT. All data are presented as mean ± SD, Kruskal‐Wallis and Dunn's multiple comparison tests, *p ≤ 0.05 and **p ≤ 0.01.

To determine whether anti‐TGFβ therapy was capable of repairing existing osteolytic bone lesions in tumor‐bearing mice, we monitored changes to bone over time in the same mice using in vivo μCT. The U266 murine model of myeloma is less aggressive than the JJN3 model used above, whereby osteolytic bone lesions in U266‐bearing mice typically evolve more slowly and can take up to 5 to 6 weeks to develop (treatment schedule described in Fig. 4 *A*). Bone disease on 3D transverse (Fig. 4 *B*) and longitudinal (Fig. 4 *C*) μCT models was apparent from 6 weeks post‐tumor cell injection (W0) (Fig. 4 *B* i, iii, v; Fig. 4 *C* i, iii, v, blue arrows) and became progressively worse over the subsequent 3 weeks (W3) (Fig. 4 *B* ii; Fig. 4 *C* ii, pink arrows). U266‐bearing mice treated with Zol alone, however, had areas of both progressive lesion development (Fig. 4 *B* iii, iv; Fig. 4 *C* iii, iv, pink arrows) and lesion repair by W3 (Fig. 4 *B* iii‐iv, green arrows). Most strikingly, the Zol and 1D11 combination group did *not* show progressive bone disease, and the initial regions of bone loss were substantially repaired by week 3 (Fig. 4 *B* v, vi; Fig. 4 *C* v, vi, green arrows). When quantified, there was a significant decrease in lesion area in the combination group compared with the Zol alone (Fig. 4 *C* vii) (*p* ≤ 0.05) and vehicle (*p* ≤ 0.05) groups. However, when overall cortical thickness and BV/TV were analyzed in this model, these parameters were not significantly different between the Zol alone and combination group but *were* significantly increased compared with the U266 vehicle group (*p* ≤ 0.05, Fig. 4 *B* viii, ix).

**Figure 5:**
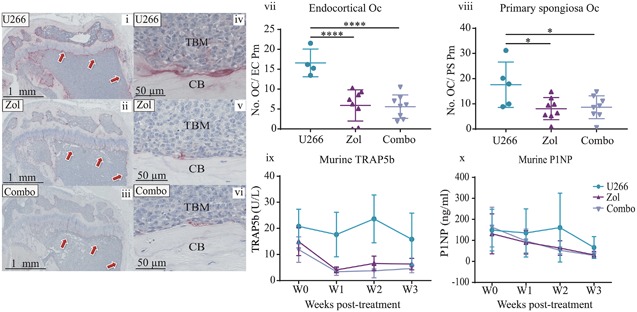
Zol and 1D11 combination therapy did not significantly decrease bone resorption or increase bone formation compared to monotherapy in the U266 model of myeloma. Representative histological images of tibias from U266‐bearing mice treated with IC (U266) (i, iv), Zol (ii, v), and Combo (iii, vi), at ×20 magnification (i–iii) and ×40 magnification (iv–vi). (vii) Number of TRAP‐positive osteoclasts on the endocortical surface and (viii) on primary spongiosa. (ix) Murine TRAP5b and (x) murine P1NP present in murine sera detected by sandwich ELISA. All data are presented as mean ± SD, ANOVA and Tukey's multiple comparison, *p ≤ 0.05 and ****p ≤ 0.0001.

To explore the mechanism of this repair, we conducted histomorphometric and serum bone turnover marker analysis. Similar to the data acquired in the JJN3 combination study (Fig. 3), U266‐bearing mice treated with Zol alone or with the combination group effectively reduced the numbers of osteoclasts by 64% (*p* ≤ 0.0001) and 66% (*p* ≤ 0.0001), respectively, on the endocortical surface compared with the U266 vehicle group (Fig. 5 i–vii). In addition, osteoclasts were significantly reduced in the primary spongiosa by 54% (*p* ≤ 0.05) in the Zol group and 51% (*p* ≤ 0.05) in the combination group compared with the U266 vehicle group (Fig. 5 viii). TRAP5b levels also correlated with histomorphometric analysis, whereby Zol alone and in combination with 1D11 reduced TRAP5b compared with the U266 vehicle group over time, but there were no differences observed between the Zol alone and the combination groups (Fig. 5 ix). Surprisingly, there were no visible osteoblasts on either of these surfaces in any of the groups, despite the obvious repair in cortical bone lesions. Similarly, there were also no differences in the serum P1NP levels between the groups (Fig. 5 x), suggesting the level of bone formation required to repair the lytic lesions was not high enough to increase systemic P1NP levels.

Overall, these data strongly demonstrate that combination therapy using TGFβ inhibition with Zol can repair existing osteolytic lesions in murine models of myeloma, a novel finding not yet reported in myeloma or other cancer‐induced bone diseases.

### TGFβ inhibition in combination with Zol does not alter JJN3 or U266 tumor burden in vivo

**Figure 6:**
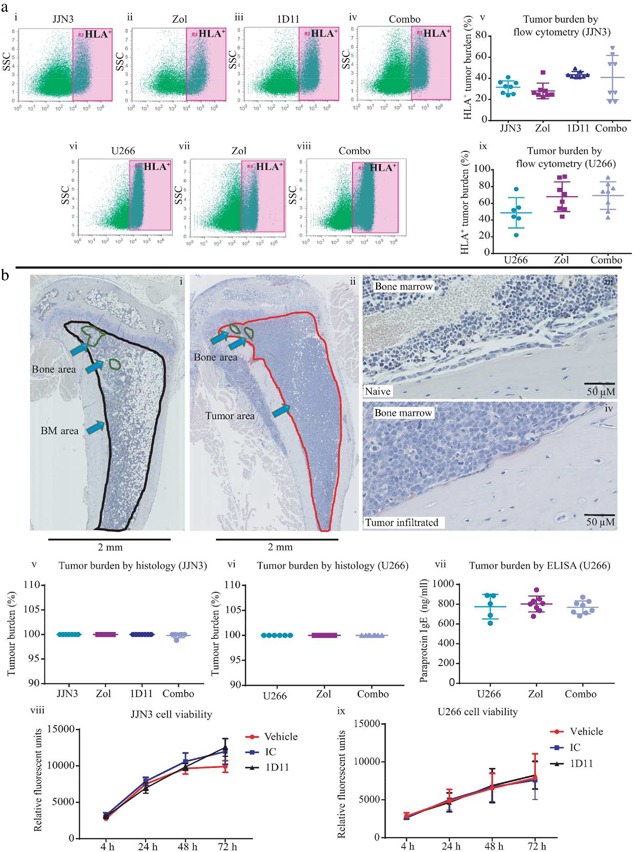
**1D11 did not inhibit the growth of JJN3 or U266 cells in vivo or in vitro.** (*A*) Representative flow cytometry dot plots showing HLA‐positive tumor cells from femora bone marrow flushes from JJN3‐bearing (i–iv) or U266‐bearing mice (vi–viii) treated with IC (JJN3 or U266) (i, vi), Zol (ii, vii), 1D11 (iii), or Combo (iv, viii), quantified as a percentage of tumor burden (v, ix). (*B*) Representative paraffin‐embedded hematoxylin‐stained bone marrow sections taken from Naïve (i ×2, iii ×40) or JJN3/U266‐bearing mice (ii ×2, iv ×40) demonstrating how percentage of tumor area is quantified using Image Scope software, normal marrow circled in black (i), bone area in circled green (i, ii), tumor area circled in red (ii). Quantification of histological tumor burden in paraffin‐embedded tibia sections taken from JJN3‐bearing mice (v) or U266‐bearing mice (vi) and treated with IC, Zol, 1D11, or Combo, quantified using Image Scope Software, as shown above, displayed as a percentage of tumor in the bone marrow compared with normal marrow. IgE paraprotein present in the sera of U266‐bearing mice at the end stage of disease, treated with IC, Zol, or Combo and analyzed by ELISA (vii). In vitro culture of JJN3 (viii) and U266 (ix) cells treated with vehicle (CRTL), IC, or 1D11 for 4 hours, 24 hours, 48 hours, and 72 hours. All data are presented as mean ± SD.

Because others have reported that TGFβ inhibition has an anti‐tumor effect in vivo in models of breast cancer and myeloma,^39^, ^40^, ^41^ we wanted to discount that any effect we observed was due to a reduction in tumor burden. Therefore, we quantified localized tumor burden in the JJN3 and U266 models by flow cytometry (Fig. 6 *A* i–ix) and histological morphology (Fig. 6 *B* i–vi). In addition, in the U266 model, we quantified systemic human IgE paraprotein by ELISA (Fig. 6 *B* vii). By all methods and in both models assessed, Zol or 1D11 monotherapies or in combination had no significant effect upon tumor burden. Therefore, this indicates that all bone effects observed in our studies were a direct effect upon bone itself and not as a result of reduced tumor burden. To confirm this, we also treated JJN3 and U266 cells in vitro with vehicle, IC, or 1D11, and no significant effects on cell viability for either cell line were observed (Fig. 6 viii, ix).

## Discussion

Multiple myeloma remains a largely incurable bone marrow cancer, which causes devastating bone disease resulting in a substantially diminished quality of life for most patients. The current standard of care for bone disease is antiresorptive therapy, usually the bisphosphonate Zol. However, antiresorptive therapy alone does not repair existing bone damage, meaning bones remain weak and are prone to fracture. Previously, bone anabolic therapy using parathyroid hormone (PTH) or anti‐sclerostin (Romosuzumab) showed improved bone outcomes in patients with metabolic disorders such as osteoporosis.^20^, ^61^, ^62^, ^63^ In addition, anti‐sclerostin therapy in preclinical models of myeloma using preventative treatment strategies have inhibited bone loss and increased bone strength,^25^, ^63^ highlighting that bone anabolic therapy could be of great benefit to patients with myeloma bone disease.

In the studies presented here, we have shown for the first time that the bone anabolic 1D11 (anti‐TGFβ antibody), when administered in combination with the antiresorptive Zol, provided a therapeutic strategy for both the prevention and, more importantly, in the treatment of myeloma‐induced bone disease. We first verified that rTGFβ inhibited osteoblast differentiation as shown previously^39^, ^58^, ^64^ and that this could be prevented using 1D11, which correlated with previous in vitro osteoblast assays using TGFβ small molecule inhibitors.^39^ In addition, we showed that 1D11 alone increased BV/TV in naïve NSG mice, supporting data by Edwards and colleagues,^29^ who looked at the effect in C57BL/6 naïve mice, and 1D11 monotherapy also prevented bone loss in the JJN3 model of myeloma, which corresponded with previous studies using the 5TGM1 model of myeloma.^46^ Most importantly, we found that when 1D11 was administered in combination with Zol, this therapeutic strategy significantly enhanced BV/TV in the JJN3 model of myeloma and treated bone disease and repaired osteolytic lesions in the U266 model of myeloma, a finding not yet reported in the literature. In addition, this effect was not mediated by a reduction in tumor burden, which did not alter after 1D11/combination treatment groups, consistent with other findings in myeloma.^46^ However, this lack of effect upon tumor burden is somewhat contradictory to some other studies in myeloma^38^, ^39^ and breast cancer models,^65^, ^66^ which did observe an anti‐tumor effect. To confirm whether 1D11 had a direct anti‐tumor effect on JJN3 or U266 cells, we treated them with 1D11 in vitro and observed no effect on cell viability for either cell line. Therefore, 1D11 does not directly influence tumor growth in vitro or in vivo, and these discrepancies with previous studies in anti‐tumor effects may be due differential regulation of factors implicated in tumor cell proliferation, including PTHrP,^41^ IL‐6,^37^ or Notch,^67^ that are controlled by TGFβ signaling, which may vary between tumor cell types and mouse strains. In addition, others have observed that the anti‐tumor effects of 1D11 may be dependent on the tumor location,^68^ the presence of other cells,^68^, ^69^, ^70^ disruption of key signaling pathways,^71^ and when 1D11 treatment commences.^69^ Therefore, there are potentially a number of reasons why we did not observe an anti‐tumor effect in our in vivo studies.

A potential limitation of our studies is they were all performed in immunocompromised mice. However, the rationale for using these models was based on the marked destructive osteolytic bone lesions that both JJN3 and U266 models feature^51^ compared with the immune‐competent 5TGM1 model, which has a less pronounced bone disease.^72^ In addition, to monitor lesion repair, a larger therapeutic window was required from onset of established osteolytic lesions to the end stage of disease. In the 5TGM1 model, this is only a few days (17 to 21 days post‐5TGM1 cell injection),^72^ whereas in the U266 model used here, it was for approximately 3 weeks (6 to 9 weeks post‐U266 cell injection). Therefore, this provided us with a larger treatment window to clearly determine the effects of combined 1D11 and Zol treatment on bone repair. In addition, Zol and 1D11 (as well as SD‐208, a small inhibitor to TGFβ receptor type 1) have previously been used in immune‐competent mice. For example, Edwards and colleagues^29^ have assessed 1D11 in naïve mice, and Nyman and colleagues^46^ have assessed 1D11 in the 5TGM1 model. Similarly, we have previously assessed the effects of Zol in immunocompromised mice^51^, ^73^ and shown similar effects on bone to immune‐competent mice.^74^ Therefore, we would expect to observe similar effects on bone in immune‐competent animals treated with combined 1D11 and Zol.

Our studies clearly demonstrate the benefits of combined 1D11 and Zol treatment on bone, yet the exact mechanism by which the repair of bone occurs remains unclear. Monotherapy of Zol or 1D11 in the JJN3 model of MM resulted in a reduction in osteoclasts, consistent with previous findings by Mohammed and colleagues^75^ and Edwards and colleagues^29^ using SD‐208/1D11 in naïve mice. In the 1D11 monotherapy group, this was potentially mediated by a reduction in IL‐6 levels, a finding described previously when SD‐208 blocked TGFβ1‐induced secretion of IL‐6 by BMSC in vitro.^39^ Nevertheless, in our in vivo studies we were unable to identify an increase in osteoblast numbers or P1NP sera levels (markers of bone formation), despite clear enhancement of bone formation. We suspect that this may be because our euthanization time points did not coincide with the precise time points at which osteoblast numbers, or biomarker levels, were increased, as well as lack of sensitivity in the sera analysis. In addition, when early and late osteocyte patterns were examined by anti‐sclerostin and anti‐E11 immunohistochemical staining, no differences were found after 1D11 treatment (data not shown), and sclerostin sera levels were also unchanged between the groups (data not shown), highlighting that there were no significant changes in osteocytes after 1D11 treatment. Studies by Nyman and colleagues^46^ did observe an increase in osteoblasts after TGFβ inhibition, but their studies differed to ours in mouse strain (C57BL/KalwRij), cell line (5TGM1), and inhibitor (SD‐208), which could have affected the osteoblast analysis. Therefore, we strongly believe that the additive effect observed in both myeloma models *is* due to both an inhibitory effect on osteoclasts and a substantial anabolic effect on osteoblastic bone formation. The latter is likely to have occurred early after treatment and thus has not been captured at the end stage of disease when tumor burden is high.

Nevertheless, the overall findings described here show a highly translational approach for the use of bone anabolic and antiresorptive therapy to prevent and repair myeloma‐induced bone disease, particularly as anti‐TGFβ therapy using a humanized antibody (GC1008) is currently in clinical trials in breast cancer (NCT01401062).^44^, ^45^ Furthermore, we are now exploring the optimum sequencing approach of bone anabolic and antiresorptive therapies, given promising data emerging from clinical trials in patients with osteoporosis.^76^, ^77^ We strongly believe that combination therapy delivered in the optimum sequence has the potential to substantially improve patient prognosis by reducing fracture risk, reducing pain, increasing functional status, and thus providing a very considerable overall improvement in quality of life for those with this devastating aspect of myeloma.

## Disclosures

All authors state that they have no conflicts of interest.
